# Dihydroisocoumarins and Dihydroisoflavones from the Rhizomes of *Dioscorea collettii* with Cytotoxic Activity and Structural Revision of 2,2′-Oxybis(1,4-di-tert-butylbenzene)

**DOI:** 10.3390/molecules26175381

**Published:** 2021-09-04

**Authors:** Songsong Jing, Zhuo Qu, Chengcheng Zhao, Xia Li, Long Guo, Zhao Liu, Yuguang Zheng, Wenyuan Gao

**Affiliations:** 1 Traditional Chinese Medicine Processing Technology Innovation Center of Hebei Province, Hebei University of Chinese Medicine, Shijiazhuang 050200, China; johnson3917@163.com (S.J.); guo_long11@163.com (L.G.); liuzhao_mbb@126.com (Z.L.); 2 Tianjin Key Laboratory for Modern Drug Delivery & High-Efficiency, School of Pharmaceutical Science and Technology, Tianjin University, Tianjin 300072, China; zhaochengcheng0122@163.com (C.Z.); lixia2008@tju.edu.cn (X.L.); 3 School of Pharmacy, Ningxia Medical University, Yinchuan 750004, China; quzhuo2008@163.com; 4 Hebei Chemical & Pharmaceutical College, Shijiazhuang 050200, China

**Keywords:** *Dioscorea collettii*, chemical constituents, dihydroisocoumarins, dihydroisoflavones, structural revision

## Abstract

The investigation of the constituents of the rhizomes of *Dioscorea collettii* afforded one new dihydroisocoumarin, named (−)-montroumarin (**1a**), along with five known compounds—montroumarin (**1b**), 1,1′-oxybis(2,4-di-tert-butylbenzene) (**2**), (3*R*)-3′-*O*-methylviolanone (**3a**), (3*S*)-3′-*O*-methylviolanone (**3b**), and (*RS*)-sativanone (**4**). Their structures were elucidated using extensive spectroscopic methods. To the best of our knowledge, compound **1a** is a new enantiomer of compound **1b**. The NMR data of compound **2** had been reported but its structure was erroneous. The structure of compound **2** was revised on the basis of a reinterpretation of its NMR data (1D and 2D) and the assignment of the ^1^H and ^13^C NMR data was given rightly for the first time. Compounds **3a**–**4**, three dihydroisoflavones, were reported from the Dioscoreaceae family for the first time. The cytotoxic activities of all the compounds were tested against the NCI-H460 cell line. Two dihydroisocoumarins, compounds **1a** and **1b,** displayed moderate cytotoxic activities, while the other compounds showed no cytotoxicity.

## 1. Introduction

*Dioscorea collettii* HK. *f*. (Dioscoreaceae) is a perennial herbaceous plant of the *Dioscorea* genus that is widely distributed in Southwest China, Myanmar, and India. The rhizomes of *D*. *collettii* have been used clinically in combination with its sister species *D*. *collettii* HK. *f*. var. *hypoglauca* in the treatment of gouty arthritis, hyperuricemia, hyperlipidemia, inflammation, and tumors in China as a traditional medicine [1,2,3,4,5]. Previous studies have revealed that the major active constituents of *D*. *collettii* are steroidal saponins [6,7]. An unusual tricyclic diarylheptanoid derivative with thirty-four compounds has been reported on the basis of the previous work of our group [8,9]. Pharmacological studies have demonstrated that steroidal saponins of the *Dioscorea* genus have significant tumor-suppressive activities in human cancer cells [10,11]. In our previous study, tsaokoarylone, a diarylheptanoid obtained from *D*. *collettii*, also showed strong cytotoxic activity against the NCI-H460 cell line [8]. However, to date, the chemical constituents of *D*. *collettii* and whether its other types of compounds have significant cytotoxic activities have remained unclear.

**Figure 1:**
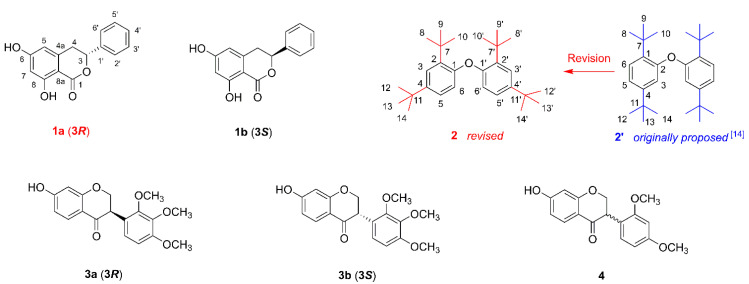
Chemical structures of compounds **1**–**4** isolated from rhizomes of *Dioscorea collettii*.

As part of our continuous search for structurally unique and biologically valuable natural products from the *Dioscorea* genus [8,9,12,13], this study aimed to investigate the phytochemical constituents and cytotoxic activity of *D*. *collettii*. In the present study, one new dihydroisocoumarin, named (−)-montroumarin (**1a**), together with five known compounds were obtained from the rhizomes of *D*. *collettii* (Figure 1). Compound **1a** is the enantiomer of the known compound montroumarin (**1b**). Compound **2** was first discovered as a natural product from the marine mollusk *Onchidium struma* [14]. The originally proposed structure of compound **2** (**2′**) (Figure 1) possessed a phenyl ether skeleton bearing four tert-butyl substituents located in the aromatic rings at the positions C-1, C-4, C-1′, and C-4′. However, during our structure elucidation of compound **2**, a closer inspection of its NMR data indicated that the originally proposed structure of compound **2** might not be correct. Herein, details of the isolation and structure elucidation of these compounds, the structural revision of compound **2**, and their cytotoxic activities against the NCI-H460 cell line are described.

## 2. Results and Discussion

### 2.1. Structural Elucidation of the Compounds

**Figure 2:**
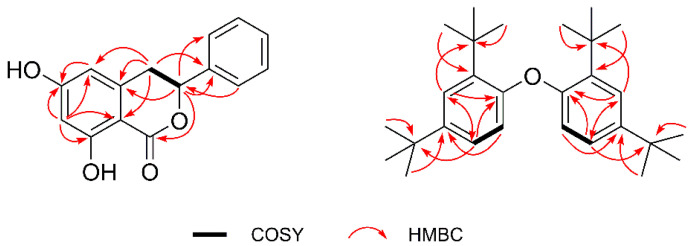
^1^H-^1^H COSY, key HMBC correlations of **1**–**2**.

Compound **1** (**1a**/**1b**) was obtained as a colorless solid. Its molecular formula was elucidated to be C_15_H_12_O_4_ by HR-ESI-MS at *m/z* 279.0626 for [M + Na]^+^ (calculated. for C_15_H_12_O_4_Na, 279.0633), with ten degrees of unsaturation. The ^13^C NMR data (Table 1) in combination with analysis of the HSQC spectrum for **1** showed the presence of one carbonyl carbon, nine olefinic or aromatic carbons, one oxymethine, and one methylene. In its ^1^H NMR spectrum, the five aromatic proton signals between *δ*_H_ 7.48 and 7.36 (*δ*_H_ 7.48 (2H, d, *J* = 7.5 Hz), 7.39 (2H, t, *J* = 7.5 Hz), and 7.36 (1H, t, *J* = 7.0 Hz)) indicated the presence of one mono-substituted benzene ring. Two meta-coupled aromatic signals at *δ*_H_ 6.26 (1H, br s) and 6.24 (1H, *J* = 1.8 Hz) suggested that **1** possessed one 1,2,3,5-tetrasubstituted aromatic ring. Additionally, one oxymethine proton at *δ*_H_ 5.56 (1H, dd, *J* = 12.0, 3.0 Hz) and one methylene group at *δ*_H_ 3.21 (1H, dd, *J* = 16.3, 12.2 Hz) and 3.06 (1H, dd, *J* = 16.4, 3.2 Hz) were observed in the ^1^H NMR spectrum, perfectly matching the above ^13^C NMR data. In addition to the two aromatic rings and one carbonyl, the remaining 1 degree of unsaturation suggested the existence of a one ring structure. All ^1^H and ^13^C chemical shifts for **1** were essentially identical to those observed for montroumarin [15]. Based on the interpretation of the HMBC and ^1^H-^1^H COSY spectra (Figure 2) as well as a comparison with the literature data, the planar structure of **1** was characterized as 6, 8-dihydroxy-3-phenyl-3, 4-dihydroisocoumarin.

**Figure 3:**
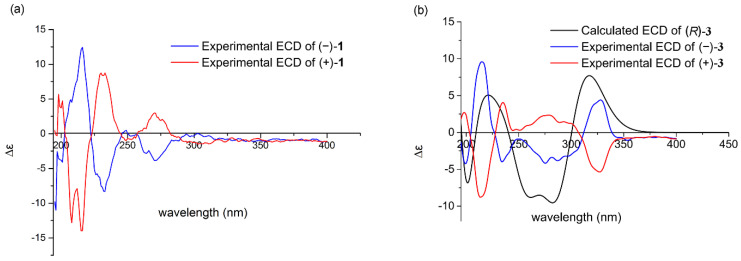
ECD spectrum of compound **1** (**a**) and experimental and calculated ECD spectra of **3** (**b**).

Due to having a single C-3 stereogenic center, **1** might exist in two potential configurations, (3*R*)-**1** and (3*S*)-**1**, and only the 3*S*-form (montroumarin) has been isolated previously [15]. Subsequent chiral HPLC separation on **1** afforded compounds **1a** and **1b**, a pair of enantiomers, in a ratio of 15:85 (Supplementary materials Figure S8), showing that **1** is a scalemic mixture. The opposite optical rotations and mirror-like electronic circular dichroism (ECD) spectra confirmed their enantiomeric relationship (Figure 3). The absolute configuration of **1a** and **1b** was determined by comparing their ECD spectra and optical rotations with those of montroumarin [15] and other similar dihydroisocoumarin derivatives [16]. The absolute configuration of (+)-**1** (**1b**) was established as *S* on the basis of the strong positive Cotton effect (CE) at 233 nm in its ECD spectrum (Figure 3), which is consistent with that of montroumarin. However, (−)-**1** (**1a**) showed a strong negative CE at 233 nm; thus, its absolute configuration was deduced to be *R*. A comparison of the optical rotations of **1a** and **1b** with that of montroumarin also supported the above conclusions. Thus, the structure of compound **1a** was defined and named as (−)-montroumarin, and **1b** was identified as the known compound montroumarin (see Figure 1).

Compound **2** was purified as a pale-yellow oil. The ^1^H NMR spectrum (Table 2) of **2** in CDCl_3_ showed signals attributable to an ABX-type aromatic ring at *δ*_H_ 7.54 (1H, d, *J* = 8.6 Hz, H-6),7.36 (1H, t, *J* = 2.5 Hz, H-3),and 7.13 (1H, dd, *J* = 8.6, 2.5 Hz, H-5), which indicated the presence of a 1,2,4-trisubstituted benzene ring [17,18]. In addition, two sets of non-equivalent signals each containing three methyl groups at *δ*_H_ 1.33 (9H, s, H-8/9/10) and 1.28 (9H, s, H-12/13/14) were also observed. The ^13^C NMR and HSQC spectra displayed 12 carbon resonances, which were classified as eight olefinic or aromatic carbons (*δ*_C_ 147.8, 147.8, 147.2, 138.7, 138.6, 124.6, 124.1, and 119.3), two quaternary carbons (*δ*_C_ 35.0 and 34.7), and two methyl carbons (*δ*_C_ 31.6 and 30.4). The ^1^H and ^13^C NMR data in CDCl_3_ (Figure S15) for compound **2** were highly consistent with the experimental data for 2, 2′-oxybis (1, 4-di-tert-butylbenzene) (**2′**), suggesting that **2** was the same substance isolated by Sun B-N and coworkers [14]. The molecular formula of compound **2′** is C_28_H_42_O and the structure of **2′** was originally identified as a phenyl ether derivative bearing four tert-butyl substituents located in the aromatic rings at the positions C-1, C-4, C-1′, and C-4′ (Figure 1). We subsequently performed 2D NMR experiments on **2**. Careful examination of the HMBC spectrum (Figure 2) of **2** revealed a strong HMBC correlation from H-5 (*δ*_H_ 7.13) to C-1 (*δ*_C_ 147.8), which is apparently inconsistent with structure **2′** (from H-2 to C-5 of **2′**) but consistent with **2**. This key HMBC correlation indicated that the structure elucidation of **2′** is clearly incorrect. The ^13^C NMR data (*δ*_C_ 147.8, 147.8, 138.7, and 138.6) and the molecular formula of **2′** indicated that **2** was a dimer. In the HMBC spectrum, the correlation from the three equivalent methyl groups signals at *δ*_H_ 1.33 (9H, H-8/9/10) to the quaternary carbon signal at *δ*_C_ 35.0 (C-7) suggested a direct connection between C-8/9/10 (*δ*_C_ 30.4) and C-7, forming one tert-butyl substituent, which was further confirmed by the HMBC correlations from H-8 (*δ*_H_ 1.33, 9H) to C-9/10 (*δ*_C_ 30.4). This tert-butyl substituent attached to C-2 (*δ*_C_ 138.6) of the aromatic ring was clarified by the correlation from H-8/9/10 to C-2 in the HMBC spectrum. Similar, the HMBC correlations from H-12/13/14 (*δ*_H_ 1.28, 9H) to C-11 (*δ*_C_ 34.7) and C-4 (*δ*_C_ 147.2) suggested a connection between C-12/13/14 (*δ*_C_ 31.6) and C-11, forming another tert-butyl substituent attached to C-4 of the aromatic ring. The ^1^H-^1^H COSY correlation (Figure 2) of H-5/H-6 (*δ*_H_ 7.54) and the HMBC correlations of H-5/C-11 and H-6/C-4 elucidated the connectivity of C-4/C-5/C-6. The linkage between C-2 and C-3 was verified by the HMBC correlation from H-3 (*δ*_H_ 7.36) to C-7. The HMBC correlations from H-5 to C-3 (*δ*_C_ 124.6) and H-3 to C-5 (*δ*_C_ 124.1), together with the C-3 and C-5 located at the meta-position of the aromatic ring, indicated the connectivity of C-2/C-3/C-4/C-5/C-6. The established linkage of C-2/C-3/C-4/C-5/C-6 as well as the HMBC correlations from H-3 and H-5 to C-1 (*δ*_C_ 147.8) constructed the aromatic ring. Considering the downfield chemical shift of C-1, and the molecular formula required for **2**, C-1 and C-1′ should be linked to the remaining one oxygen atom to form a dimer. The structure of **2**, shown in Figure 1, was identified as 1,1′-oxybis(2,4-di-tert-butylbenzene). 

This explained well the strong HMBC correlation from H-5 (*δ*_H_ 7.13) to C-1 (*δ*_C_ 147.8), which is apparently inconsistent with structure **2′**. In addition, all the HMBC correlations reported for **2′** [14], including the weak ^4^*J*_C,H_ long-range HMBC correlation from H-6 (*δ*_H_ 7.54) to C-7 (*δ*_C_ 35.0) [19,20], were also consistent with structure **2**. Moreover, compound **2** might be a naturally occurring dimer of 2,4-Di-tert-butylphenol (2,4-DTBP), which is a common secondary metabolite produced by various groups of organisms [21]. Therefore, we compared the chemical shifts of compounds **2**, **2′**, and 2,4-DTBP and found that the structure elucidation of **2** was more reasonable than that of **2′** [22,23] (Figure S15). When running a ^13^C spectrum prediction for compounds 2,4-DTBP and **2′**, it is trivial to see that the ^13^C-data of 2,4-DTBP are consistent with structure **2**, whereas in the case of **2′** there is a massive inconsistency. By searching SciFinder, we found that the structure of **2** was not reported in any literature or patents but only had one commercial source. This was the first report of its detailed structure elucidation based on 1D and 2D NMR spectroscopy data.

Compound **3** (**3a**/**3b**) was obtained as a colorless needle. The results of a comparison between the ^1^H, ^13^C NMR data of **3** and those reported by Guimarães et al. [24] suggested that the planar structure of **3** was 3′-*O*-methylviolanone. Compound **3** was optically inactive, suggesting that it was a racemic mixture. Both of the two configurations (3*R*)-3 and (3*S*)-3 have been reported previously [25,26], but the ECD spectra of (3*R*/3*S*)-**3** and the specific rotation of (3*R*)-**3** have remained undefined. Subsequent chiral resolution of **3** (Figure S18) afforded a pair of enantiomers, **3a** and **3b**, and they displayed almost mirror-image ECD curves that showed opposite CEs at 193, 210, 230, 272, and 325 nm. The respective absolute configurations of (−)-**3** and (+)-**3** (**3a** and **3b**) were defined as (3*R*) and (3*S*) via a comparison of their experimental and calculated ECD spectra (Figure 3).

Compound **4** was also obtained as a colorless needle. A comparison of the ^1^H and ^13^C NMR data of **4** with those of compound **3** showed that their structures are closely related, suggesting that **4** is also a dihydroisoflavone analogue. Through a comparison of its NMR data with those reported [27], the planar structure of **4** was identified as sativanone. Similarly to compound **3**, **4** was also optically inactive, suggesting that it was a racemate. Both of the two configurations (3*R*)-**4** and (3*S*)-**4**, including their ECD spectra and the specific rotations, have been reported [28,29]. Thus, compound **4** was identified as (*RS*)-sativanone.

### 2.2. Cytotoxic Activity against NCI-H460 Cell Line

All the isolated compounds **1**–**4** were further evaluated for their cytotoxicity against the human lung cancer NCI-H460 cell line (Table 3). The results indicated that compounds **1a** and **1b**, two dihydroisocoumarins, possess moderate cytotoxic activities, with IC_50_ values of 33.37 and 32.06 μM, respectively. Compounds **2**–**4** showed no activity, with an IC_50_ > 50 μM.

## 3. Materials and Methods

### 3.1. General Information

One-dimensional- and two-dimensional-NMR spectra were obtained from a Bruker Avance III 600 MHz spectrometer (Bruker BioSpin Inc., Zurich, Switzerland). Optical rotations were measured with an MCP 200 Modular Circular Polarimeter (Anton Paar, Graz, Austria). HRESIMS spectrum was obtained from a MicrOTOF-Q II ESI mass spectrometer (Bruker, Bremen, Germany). UV spectrum was obtained from a Hitachi U-3900 UV-visible spectrophotometer (Hitachi High-Tech Science Corporation, Tokyo, Japan). ECD spectra were recorded by a MOS-500 Circular Dichroism spectropolarimeter (Bio-Logic, Grenoble, France). Semi-preparative HPLC was performed on an LC-6A (Shimadzu, Kyoto, Japan) equipped with preparative YMC Pack ODS-A column (250 × 20 mm, 5 μm, YMC, Kyoto, Japan). A Daicel Chiralpak AD-H (250 × 4.6 mm, 5 μm, Daicel, Tokyo, Japan) was used for the chiral separations. Column chromatography (CC) was carried out over silica gel (100–200 and 200–300 mesh, Qingdao Haiyang Chemical Co., Ltd., Qingdao, China), Sephadex LH-20 (Amersham Pharmacia Biotech AB, Uppsala, Sweden), and ODS RP-C18 (40–63 μm, YMC, Kyoto, Japan). Thin Layer Chromatography (TLC) was performed on pre-coated silica gel GF-254 (Qingdao Marine Chemical Factory, Qingdao, China).

### 3.2. Materials

The rhizomes of *D*. *collettii* were collected from Mount Emei, Leshan City, Sichuan province, China, and authenticated by Prof. Wenyuan Gao (School of Pharmaceutical Science and Technology, Tianjin University). A voucher specimen (ID: 245020328) was deposited in the School of Pharmaceutical Science and Technology, Tianjin University, Tianjin, China.

### 3.3. Extraction and Isolation

The air-dried rhizomes of *D*. *collettii* (16.2 kg) were extracted three times with 90% aqueous ethanol and three times with 60% aqueous ethanol under reflux (30 L, each for 2 h). After removal of the solvent under reduced pressure, the residue was combined and suspended in water to a final volume of 10 L, and then sequentially partitioned with petroleum ether (PE, 60–90 °C), ethyl acetate (EtOAc), and n-butyl alcohol (n-BuOH).

The EtOAc extract (305.0 g) was subjected to silica gel column chromatography (CC) eluted with CH_2_Cl_2_-MeOH gradient (10:0 to 0:10, *v/v*) to afford 19 fractions (A–S). Fraction G was purified by Sephadex LH-20 CC eluted with CH_2_Cl_2_-MeOH (1:1) to yield 5 fractions (G1–G5). Fraction G2 was exposed to silica gel CC eluted with PE-EtOAc gradient (1:0 to 7:3, *v/v*) followed by Sephadex LH-20 CC eluted with CH_2_Cl_2_-MeOH (1:1), and finally purified by semi-preparative HPLC (YMC C18, 250 × 20 mm, 5 μm, 10 mL/min) eluted with 65% aqueous MeOH to yield compound **1** (15 mg, *t*_R_ = 8 min). Fraction G4 was isolated by Sephadex LH-20 CC eluted with CH_2_Cl_2_-MeOH (1:1) to yield 51 subfractions (Fr.1–Fr.51). Fr.28–Fr.30 were submitted to silica gel CC eluted with PE-EtOAc (75:25, *v/v*) and then purified by Sephadex LH-20 CC eluted with CH_2_Cl_2_-MeOH (1:1) to obtain compound **3** (16 mg). Fr.31–Fr.39 was applied on silica gel CC eluted with PE-EtOAc (8:2, *v/v*) to obtain compound **4** (7 mg). Fraction L was chromatographed over silica gel CC with PE-EtOAc solvent system with increasing polarity to afford 3 fractions (L1–L3). Fraction L1 was isolated by Sephadex LH-20 CC eluted with CH_2_Cl_2_-MeOH (1:1) to obtain compound **2** (9 mg).

Compound **1** was separated using chiral-phase HPLC (AD-H column, n-hexane/ethanol, 70:30, flow rate: 1.0 mL/min) to afford **1b** (*t*_R_ = 10.0 min) and **1a** (*t*_R_ = 11.9 min). Compound **3** was further resolved using chiral-phase HPLC (AD-H column, n-hexane/ethanol, 85:15, flow rate: 1.0 mL/min) to obtain **3b** (*t*_R_ = 8.2 min) and **3a** (*t*_R_ = 9.2 min).

#### 3.3.1. (−)-Montroumarin (**1a**)

Colorless solid; ${\left[\alpha \right]}_{D}^{20}$ − 93.65 (c 0.20, MeOH); UV (MeOH) λmax 195, 217, 271, 303 nm; ^1^H-NMR (600 MHz, CD_3_OD) and ^13^C-NMR (150 MHz, CD_3_OD) spectroscopic data, see Table 1; HR ESI-TOF MS *m/z* 279.0626 [M + Na]^+^ (calcd. for C_15_H_12_O_4_Na 279.0633).

#### 3.3.2. Montroumarin (**1b**)

Colorless solid; ${\left[\alpha \right]}_{D}^{20}$ + 71.72 (c 0.20, MeOH); ^1^H-NMR (600 MHz, CD_3_OD) and ^13^C-NMR (150 MHz, CD_3_OD) spectroscopic data, see Table 1; HR ESI-TOF MS data, see compound **1a**.

#### 3.3.3. 1,1′-Oxybis(2,4-di-tert-butylbenzene) (**2**)

Pale-yellow oil; ^1^H-NMR (600 MHz, CDCl_3_) and ^13^C-NMR (150 MHz, CDCl_3_) spectroscopic data, see Table 2; HR ESI-TOF MS *m/z* 395.3373 [M + H]^+^ (calcd. for C_28_H_43_O 395.3314).

#### 3.3.4. (3*R*)-3′-*O*-Methylviolanone (**3a**)

Colorless needle; ${\left[\alpha \right]}_{D}^{20}$ − 39.17 (c 0.20, MeOH); ^1^H-NMR (600 MHz, C_5_D_5_N) *δ*: 4.79 (1H, t, *J* = 11.3 Hz, H-2ax), 4.44 (1H, dd, *J* = 11.7 Hz, 5.4 Hz, H-3ax), 4.60 (1H, dd, *J* = 10.8 Hz, 5.5 Hz, H-2eq), 8.23 (1H, d, *J* = 8.6 Hz, H-5), 6.93 (1H, dd, *J* = 8.6 Hz, 2.2 Hz, H-6), 6.84 (1H, d, *J* = 2.2 Hz, H-8), 6.71 (1H, d, *J* = 8.5 Hz, H-5′), 7.03 (1H, d, *J* = 8.5 Hz, H-6′), 3.84 (1H, s, 2′-OMe), 3.92 (1H, s, 3′-OMe), 3.72 (1H, s, 4′-OMe). ^13^C-NMR (150 MHz, C_5_D_5_N) *δ*: 72.1 (C-2), 49.1 (C-3), 191.6 (C-4), 130.4 (C-5), 112.1 (C-6), 166.5 (C-7), 104.2 (C-8), 164.9 (C-8a), 115.6 (C-4a), 123.1 (C-1′), 153.1 (C-2′), 143.4 (C-3′), 154.5 (C-4′), 108.7 (C-5′),125.4 (C-6′), 61.3 (C-2′-OMe), 60.9 (C-4′-OMe), 56.4 (C-3′-OMe); HR ESI-TOF MS *m/z* 353.0998 [M + Na]^+^ (calcd. for C_18_H_18_O_6_Na 353.1001).

#### 3.3.5. (3*S*)-3′-*O*-Methylviolanone (**3b**)

Colorless needle; ${\left[\alpha \right]}_{D}^{20}$ + 28.33 (c 0.20, MeOH); ^1^H-NMR (600 MHz, C_5_D_5_N), ^13^C-NMR (600 MHz, C_5_D_5_N) spectroscopic data and HR ESI-TOF MS data, see compound **3a**.

#### 3.3.6. (*RS*)-Sativanone (**4**)

Colorless solid; ${\left[\alpha \right]}_{D}^{20}$ − 0.06 (c 0.38, MeOH); ^1^H-NMR (600 MHz, C_5_D_5_N) *δ*: 4.76 (1H, t, *J* = 11.1 Hz, H-2ax), 4.50 (1H, dd, *J* = 11.3 Hz, 5.2 Hz, H-3ax), 4.59 (1H, dd, *J* = 10.8 Hz, 5.3 Hz, H-2eq), 8.25 (1H, d, *J* = 8.6 Hz, H-5), 6.92 (1H, dd, *J* = 8.6 Hz, 2.1 Hz, H-6), 6.84 (1H, d, *J* = 2.1 Hz, H-8), 6.67 (1H, d, *J* = 2.0 Hz, H-3′), 6.58 (1H, dd, *J* = 8.3 Hz, 2.0 Hz, H-5′), 7.24 (1H, d, *J* = 8.3 Hz, H-6′), 3.63 (1H, s, 2′-OMe), 3.69 (1H, s, 4′-OMe). ^13^C-NMR (150 MHz, C_5_D_5_N) *δ*: 71.8 (C-2), 48.2 (C-3), 191.6 (C-4), 130.4 (C-5), 112.0 (C-6), 166.5 (C-7), 104.0 (C-8), 164.9 (C-8a), 115.7 (C-4a), 117.5 (C-1′), 159.4 (C-2′), 99.8 (C-3′), 161.4 (C-4′), 105.7 (C-5′), 131.7 (C-6′), 55.9 (C-2′-OMe), 55.6 (C-4′-OMe); HR ESI-TOF MS *m/z* 301.1006 [M + H]^+^ (calcd. for C_17_H_17_O_5_ 301.1076).

### 3.4. Cytotoxicity Assays

Compounds **1**–**4** were evaluated for their cytotoxic activities by the MTT method using NCI-H460 cell line. The NCI-H460 cells were seeded at a density of 1 × 104/well in a complete growth medium in 96-well plates. The cells were incubated with the test compounds for 24 h before the MTT assay. Then, a fresh solution of MTT (0.5 mg/mL) was added to each single well of the 96-well plate. The plate was incubated in a CO_2_ incubator for another 4 h. Finally, the cells were dissolved with 100 μL of DMSO and then analyzed in a multiwall plate reader with a wavelength of 570 nm.

## 4. Conclusions

The chemical investigation of *D*. *collettii* led to the isolation of six compounds (**1**–**4**), including one new dihydroisocoumarin, (−)-montroumari (**1a**). The structure of 2,2′-oxybis(1,4-di-tert-butylbenzene) (**2′**) was revised to be 1,1′-oxybis(2,4-di-tert-butylbenzene) (**2**), assisted by a careful re-examination of the structural elucidation process. All the compounds **1**–**4** were isolated from *D*. *collettii* for the first time. The isolation of compounds **3a**, **3b**, and **4** from the *Dioscorea* species has not been reported yet [30,31]. Notably, to the best of our knowledge, dihydroisoflavones have not been described from any other species in the Dioscoreaceae family. Our discovery of these dihydroisoflavones (**3a**, **3b**, and **4**) enriches the structural diversity of the Dioscoreaceae family. Dihydroisocoumarins (**1a**–**1b**) exhibited moderate cytotoxic activities against the NCI-H460 cell line, with IC_50_ values ranging from 32.06 to 33.37 μM, whereas the other compounds, including the three dihydroisoflavones (**3a**–**4**), did not show any activities at the tested concentrations (IC_50_ > 50 μM). Mounting evidence has revealed that steroidal saponins exert strong cytotoxic activities against human cancer cells, which means they could be considered as promising cytotoxic agents against human cancer cells. Dihydroisocoumarins and diarylheptanoids showed moderate cytotoxic activities against the NCI-H460 cell line. However, more selectivity studies are needed to determine whether these two types of compounds in *D. collettii* have cytotoxic activities against human cancer cells.

## Figures and Tables

**Table 1:** ^1^H-NMR (600 MHz), ^13^C NMR (150 MHz) data, and HMBC correlations of compound **1** in CD_3_OD.

No.	*δ*_H_, mult (*J* in Hz)	*δ*_C_	HMBC (H→C)
1		171.5, C	
3	5.56, dd (12.0, 3.0)	81.8, CH	C-1, C-4, C-4a, C-1′, C-2′, C-6′
4	3.21, dd (16.3, 12.2) 3.06, dd (16.4, 3.2)	36.0, CH_2_	C-3, C-4a, C-5, C-8a, C-1′
4a		143.4, C	
5	6.26, br s	108.0, CH	C-4, C-6, C-7, C-8a
6		166.4, C	
7	6.24, d (1.8)	102.4, CH	C-5, C-6, C-8, C-8a
8		165.7, C	
8a		101.7, C	
1′		140.1, C	
2′, 6′	7.48, d (7.5)	127.3, CH	C-3, C-3′, C-4′, C-5′
3′, 5′	7.41, t (7.5)	129.7, CH	C-1′, C-3′, C-5′
4′	7.36, t (7.0)	129.7, CH	C-2′, C-6′

**Table 2:** ^1^H-NMR (600 MHz), ^13^C NMR (150 MHz) data, and HMBC correlations of compound **2** in CDCl_3_.

No.	*δ*_H_, mult (*J* in Hz)	*δ*_C_	HMBC (H→C)
1 (1′)		147.8 (147.8), C	
2 (2′)		138.6 (138.7), C	
3 (3′)	7.36, t (2.5)	124.6, CH	C-7, C-11, C-5, C-1 (C-7′, C-11′, C-5′, C-1′)
4 (4′)		147.2, C	
5 (5′)	7.13, dd (8.6, 2.5)	124.1, CH	C-11, C-6, C-3, C-1 (C-11′, C-6′, C-3′, C-1′)
6 (6′)	7.54, d (8.6)	119.3, CH	C-2, C-4 (C-2′, C-4′)
7 (7′)		35.0, C	
8/9/10 (8′/9′/10′)	1.33, s	30.4, CH_3_	C-7, C-2 (C-7′, C-2′)
11 (11′)		34.7, C	
12/13/14 (12′/13′/14′)	1.28, s	31.6, CH_3_	C-11, C-4 (C-11′, C-4′)

**Table 3:** The IC_50_ value of compounds on NCI-H460 cells (μM).

Sample	IC_50_ (μM)	Sample	IC_50_ (μM)
Compound **1a**	33.37	Compound **1b**	32.06
Compound **2**	>100	Compound **3a**	57.83
Compound **3b**	59.34	Compound **4**	64.01
Cisplatin	1.27		

## Data Availability

Data are contained within the article or supplementary material.

## Supplementary Materials

The following are available online. Figure S1: ^1^H-NMR (600 MHz, CD_3_OD) spectrum of compound **1a**/**1b**, Figure S2: ^13^C-NMR (150 MHz, CD_3_OD) spectrum of compound **1a**/**1b**, Figure S3: HSQC spectrum of compound **1a**/**1b**, Figure S4: ^1^H-^1^H COSY spectrum of compound **1a**/**1b**, Figure S5: HMBC spectrum of compound **1a**/**1b**, Figure S6: HR-ESI-MS spectrum of compound **1a**/**1b**, Figure S7: UV spectrum of compound **1a**/**1b**, Figure S8: Chiral HPLC separation chromatogram of compound **1**, Figure S9: ^1^H-NMR (600 MHz, CDCl_3_) spectrum of compound **2**, Figure S10: ^13^C-NMR (150 MHz, CDCl_3_) spectrum of compound **2**, Figure S11: HSQC spectrum of compound **2**, Figure S12: ^1^H-^1^H COSY spectrum of compound **2**, Figure S13: HMBC spectrum of compound **2**, Figure S14: The enlarged HMBC spectrum of compound 2 (I, II, III, and IV), Figure S15: Structures and comparison of chemical shifts between compounds **2**, **2′**, and **2,4-DTBP** in CDCl_3_, Figure S16: ^1^H-NMR (600 MHz, C_5_D_5_N) spectrum of compound **3a**/**3b**, Figure S17: ^13^C-NMR (150 MHz, C_5_D_5_N) spectrum of compound **3a**/**3b**, Figure S18: Chiral HPLC separation chromatogram of compound **3**, Figure S19: ^1^H-NMR (600 MHz, C_5_D_5_N) spectrum of compound **4**, Figure S20: ^13^C-NMR (150 MHz, C_5_D_5_N) spectrum of compound **4**, Figure S21: ECD spectrum of compound **3**, and ECD calculation of compound **3**.

## Sample Availability

Samples of the compounds **1**–**4** are available from the authors.
