# Supplementary material for: Dihydroisocoumarins and Dihydroisoflavones from the Rhizomes of Dioscorea collettii with Cytotoxic Activity and Structural Revision of 2,2′-Oxybis(1,4-di-tert-butylbenzene)
Source: Molecules. 2021 Sep 4;26(17):5381. doi: 10.3390/molecules26175381 (PMC8433754; doi:10.3390/molecules26175381)
Supplement: Supplementary file 1 [file molecules-26-05381-s001.zip › molecules-1357413-supplementary.pdf]

Supporting Information

Dihydroisocoumarins and dihydroisoflavones from the rhizomes of *Dioscorea collettii* with cytotoxic activity and structural revision of 2, 2'-oxybis (1, 4-di-tert-butylbenzene)

Songsong Jing [1,2,†], Zhuo Qu [3,†], Chengcheng Zhao [2], Xia Li [2], Long Guo [1], Zhao Liu [1], Yuguang Zheng [1,4,*] and Wenyuan Gao [2,*]

[1] Traditional Chinese Medicine Processing Technology Innovation Center of Hebei Province, Hebei University of Chinese Medicine, Shijiazhuang 050200, China; johnson3917@163.com (S. J.); guo_long11@163.com (L.G.); liuzhao_mbb@126.com (Z. L.)

[2] Tianjin Key Laboratory for Modern Drug Delivery & High-Efficiency, School of Pharmaceutical Science and Technology, Tianjin University, Tianjin 300072, China; zhaochengcheng0122@163.com (C. Z.); lix-ia2008@tju.edu.cn (X. L.)

[3] School of Pharmacy, Ningxia Medical University, Yinchuan 750004, China; quzhuo2008@163.com (Z. Q.)

[4] Hebei Chemical & Pharmaceutical College, Shijiazhuang 050200, China

* Correspondence: pharmgao@tju.edu.cn (W.G.); zyg314@163.com (Y.Z.); Tel.: +86-022-87401895 (W.G.)

† These authors contributed equally to this work.

Contents

Figure S1: ^{1}H-NMR (600 MHz, CD_3OD) spectrum of compound **1a/1b**.

Figure S2: ^{13}C-NMR (150 MHz, CD_3OD) spectrum of compound **1a/1b**.

Figure S3: HSQC spectrum of compound **1a/1b**.

Figure S4: ^{1}H-^{1}H COSY spectrum of compound **1a/1b**.

Figure S5: HMBC spectrum of compound **1a/1b**.

Figure S6: HR-ESI-MS spectrum of compound **1a/1b**.

Figure S7: UV spectrum of compound **1a/1b**.

Figure S8 Chiral HPLC separation chromatogram of compound **1**.

Figure S9: ^{1}H-NMR (600 MHz, $CDCl_3$) spectrum of compound **2**.

Figure S10: ^{13}C-NMR (150 MHz, $CDCl_3$) spectrum of compound **2**.

Figure S11: HSQC spectrum of compound **2**.

Figure S12: ^{1}H-^{1}H COSY spectrum of compound **2**.

Figure S13: HMBC spectrum of compound **2**.

Figure S14 (I). The enlarged HMBC spectrum of compound **2** (I).

Figure S14 (II). The enlarged HMBC spectrum of compound **2** (II).

Figure S14 (III). The enlarged HMBC spectrum of compound **2** (III).

Figure S14 (IV). The enlarged HMBC spectrum of compound **2** (IV).

Figure S15. Structures and comparison of chemical shifts between compounds **2, 2'** and **2,4-DTBP** in $CDCl_3$.

Figure S16. ^{1}H-NMR (600 MHz, C_5D_5N) spectrum of compound **3a**/**3b**.

Figure S17. ^{13}C-NMR (150 MHz, C_5D_5N) spectrum of compound **3a**/**3b**.

Figure S18. Chiral HPLC separation chromatogram of compound **3**.

Figure S19. ^{1}H-NMR (600 MHz, C_5D_5N) spectrum of compound **4**.

Figure S20. ^{13}C-NMR (150 MHz, C_5D_5N) spectrum of compound **4**.

Figure S21. ECD spectrum of compound **3**

ECD calculation of compound **3**

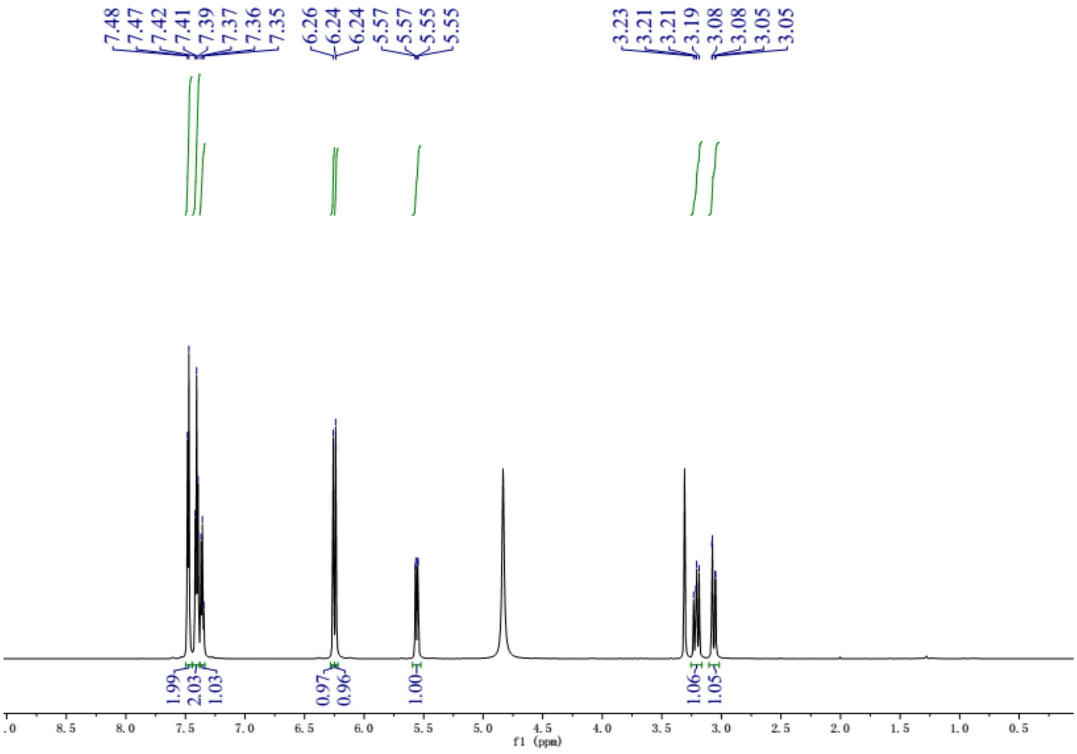

Figure S1. ^{1}H-NMR (600 MHz, CD_3OD) spectrum of compound **1a**/**1b**.

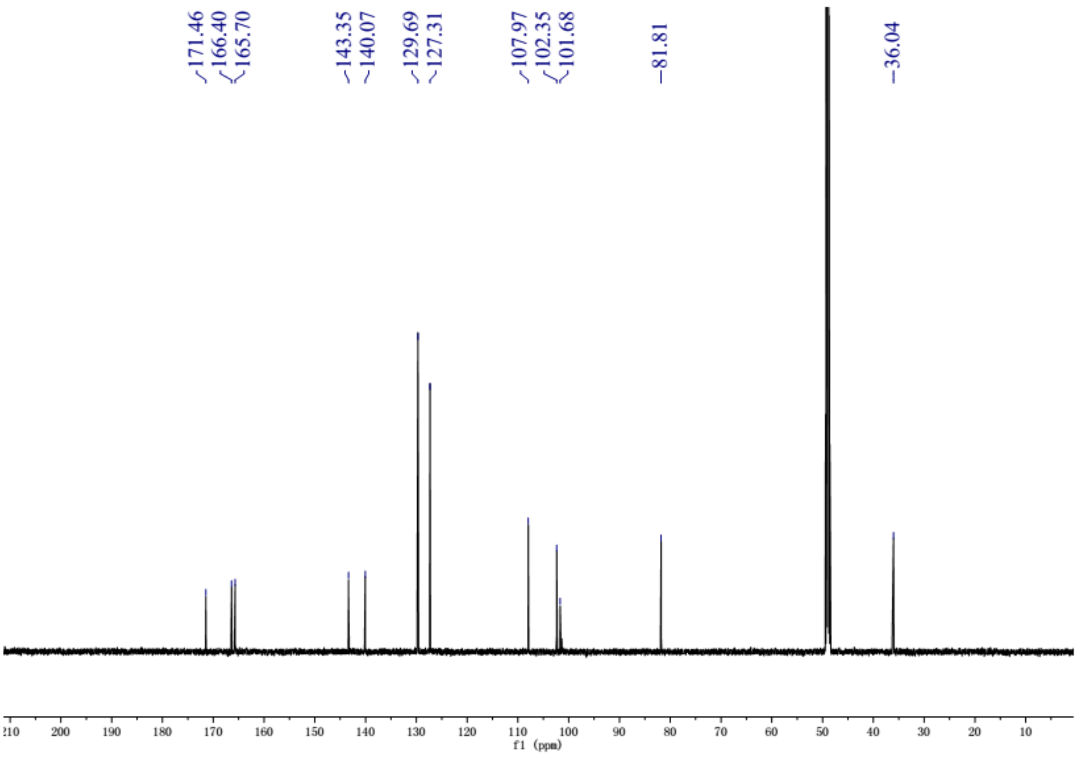

Figure S2. ^{13}C-NMR (150 MHz, CD_3OD) spectrum of compound **1a**/**1b**.

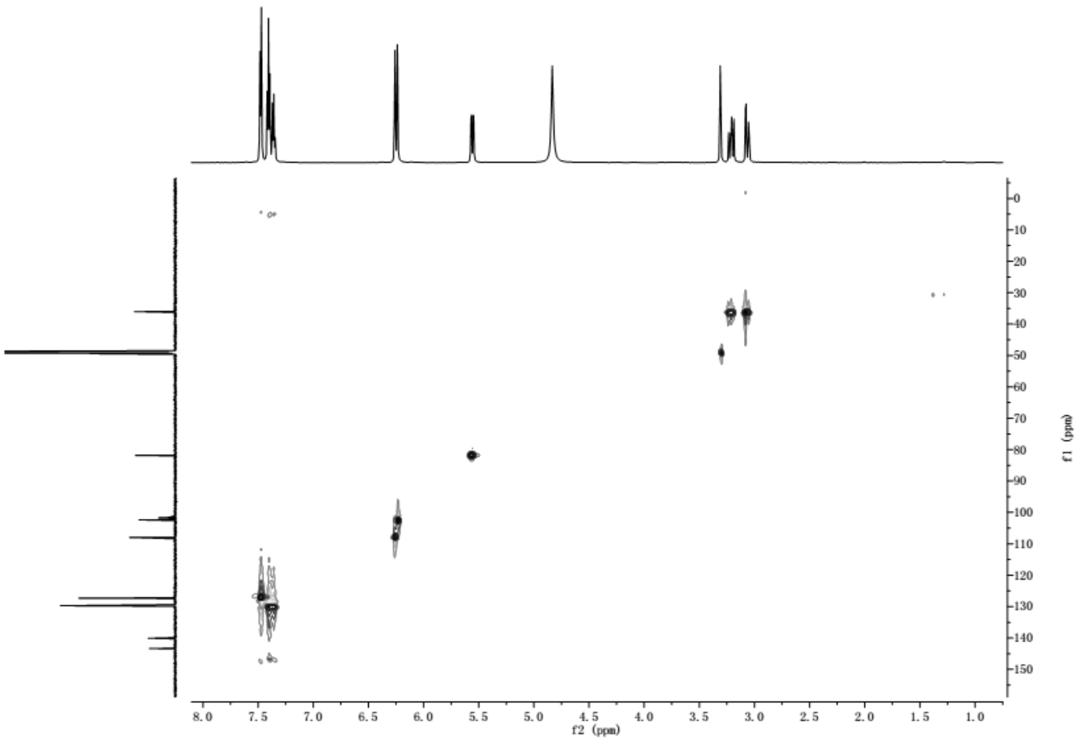

Figure S3. HSQC spectrum of compound **1a**/**1b**.

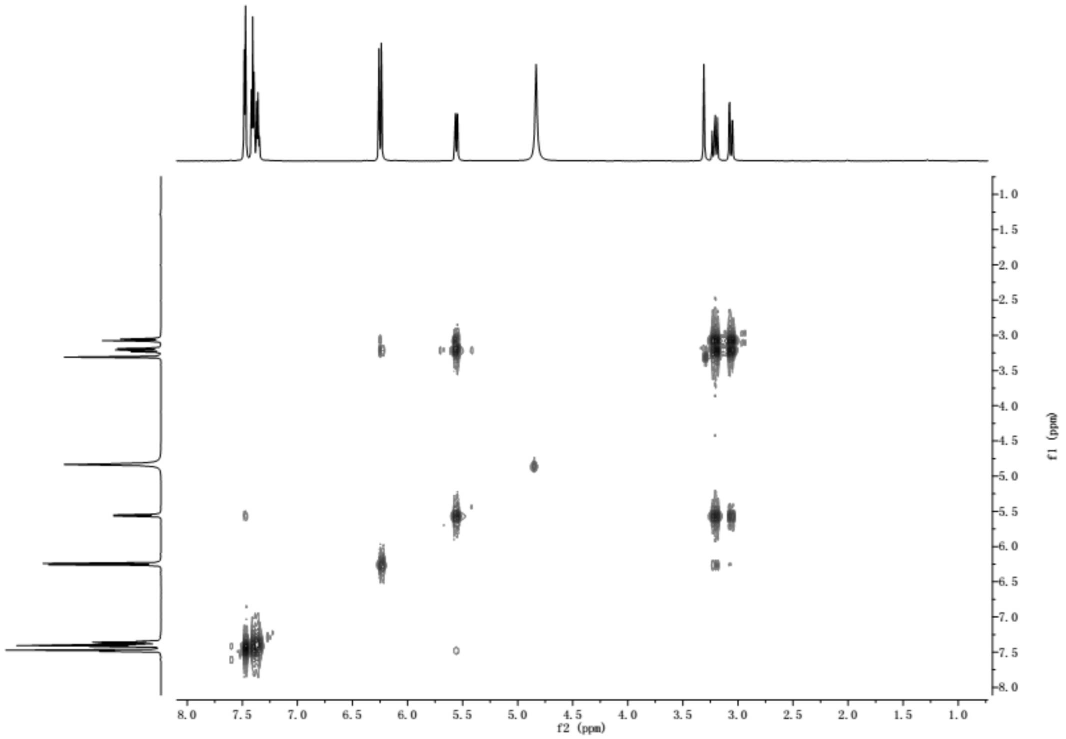

Figure S4. ^{1}H-^{1}H COSY spectrum of compound **1a/1b**.

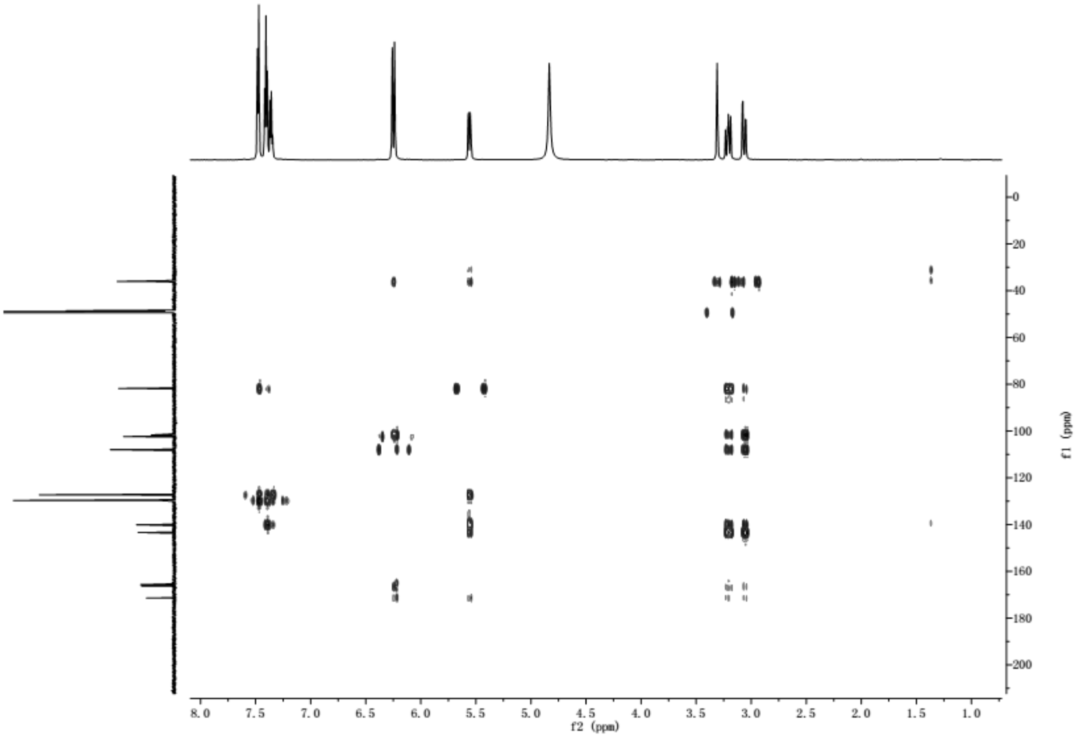

Figure S5. HMBC spectrum of compound **1a/1b**.

User Spectra

Fragmentor Voltage	Collision Energy	Ionization Mode
175	0	ESI

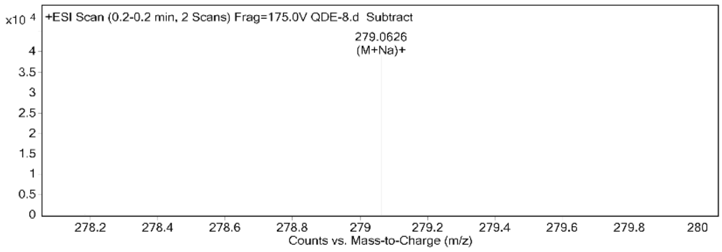

Peak List

m/z	z	Abund	Formula	Ion
239.07	1	14611.87		
257.0809	1	4990.47		
279.0626	1	39046.41	C15 H12 O4	(M+Na)+
280.0664	1	6500.46	C15 H12 O4	(M+Na)+
301.0449	1	2954.23		
301.141	1	4330.71		
353.2657	1	3101.23		
535.1358	1	7949.18		

Formula Calculator Element Limits

Element	Min	Max
C	3	60
H	0	120
O	0	30

Formula Calculator Results

Formula	CalculatedMass	CalculatedMz	Mz	Diff. (mDa)	Diff. (ppm)	DBE
C15 H12 O4	256.0736	279.0628	279.0626	0.1	0.4	10.0000

--- End Of Report ---

Figure S6. HR-ESI-MS spectrum of compound **1a**/**1b**.

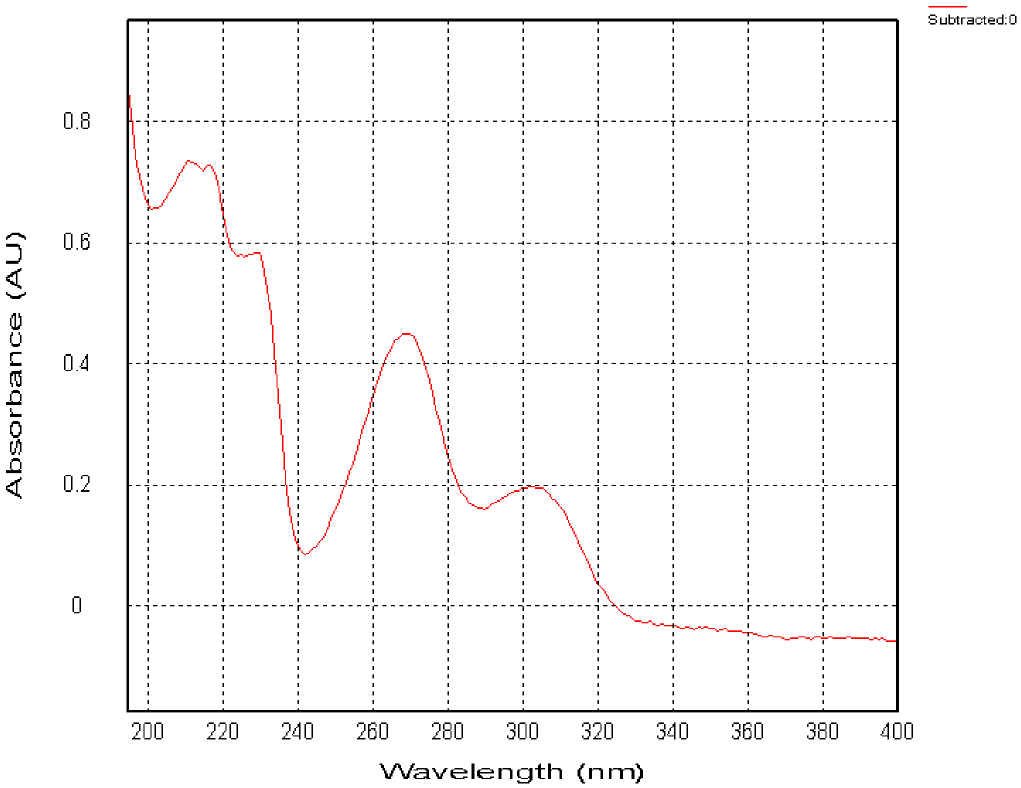

Figure S7. UV spectrum of compound **1a**/**1b**.

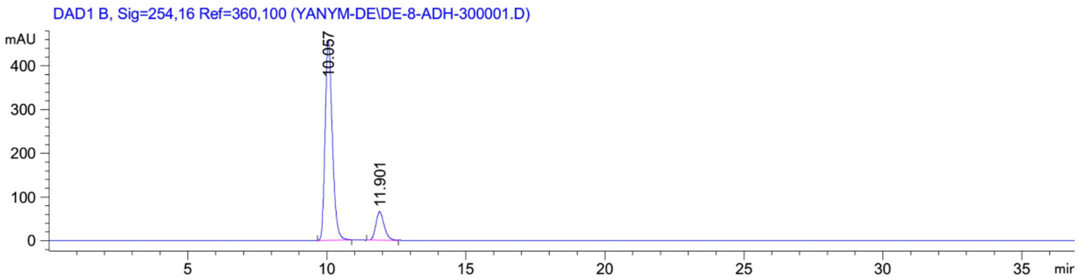

Figure S8. Chiral HPLC separation chromatogram of compound **1**.

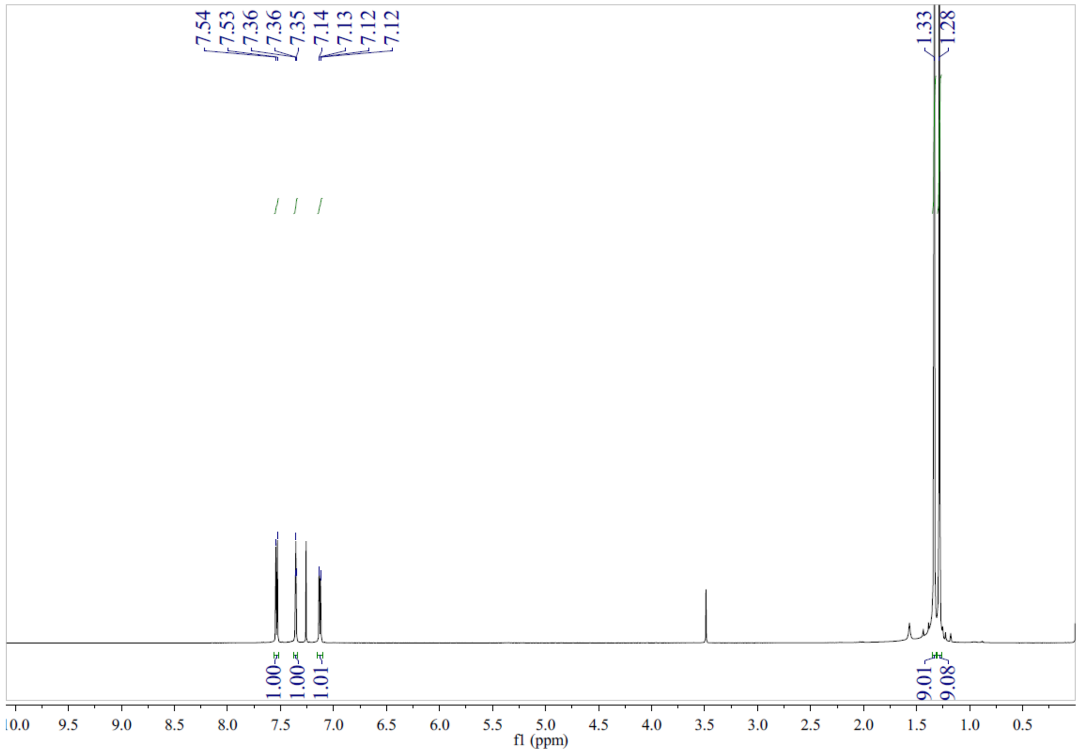

Figure S9. ^{1}H-NMR (600 MHz, $CDCl_3$) spectrum of compound **2**.

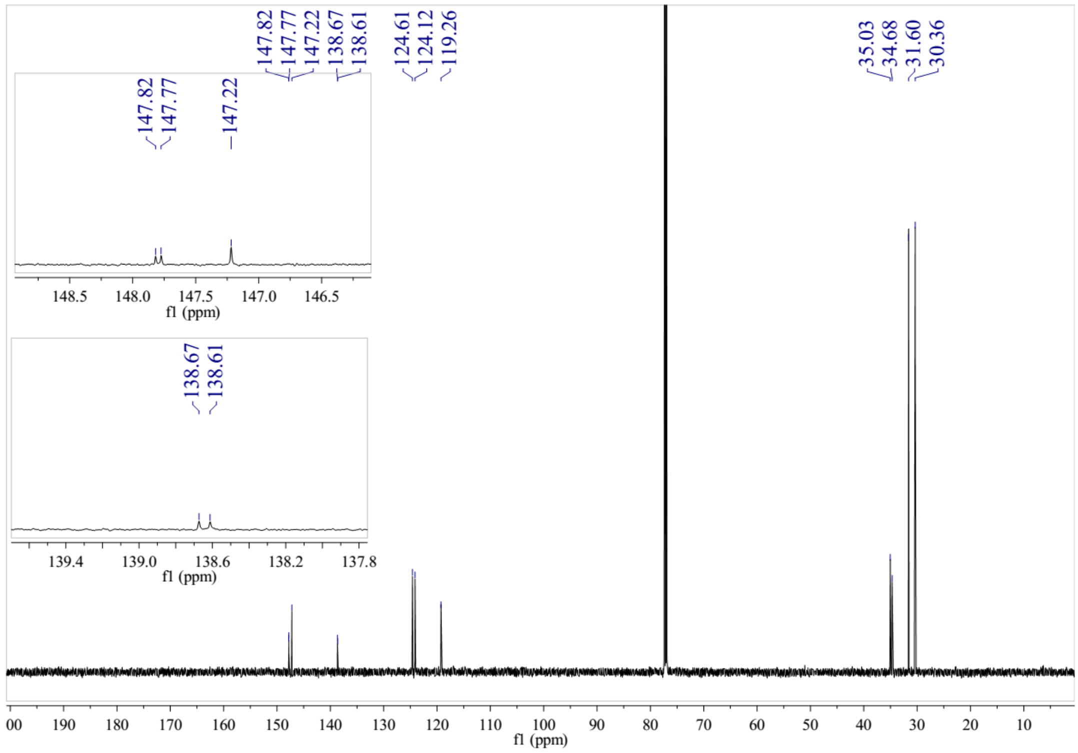

Figure S10. ^{13}C-NMR (150 MHz, $CDCl_3$) spectrum of compound **2**.

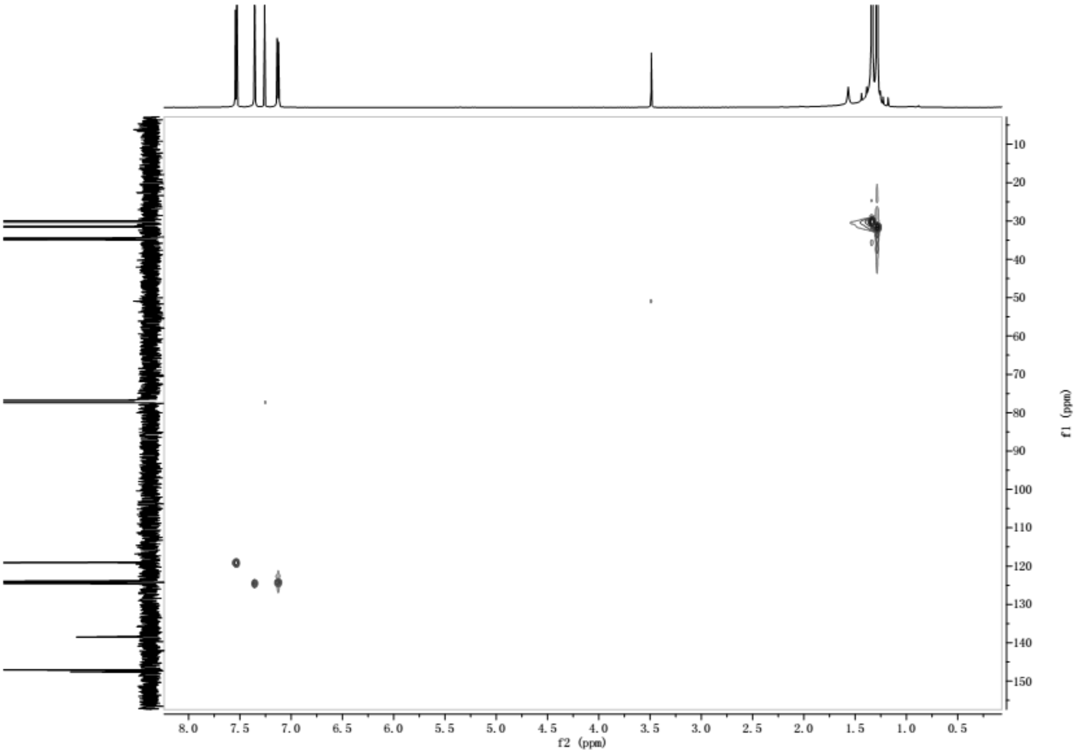

Figure S11. HSQC spectrum of compound **2**.

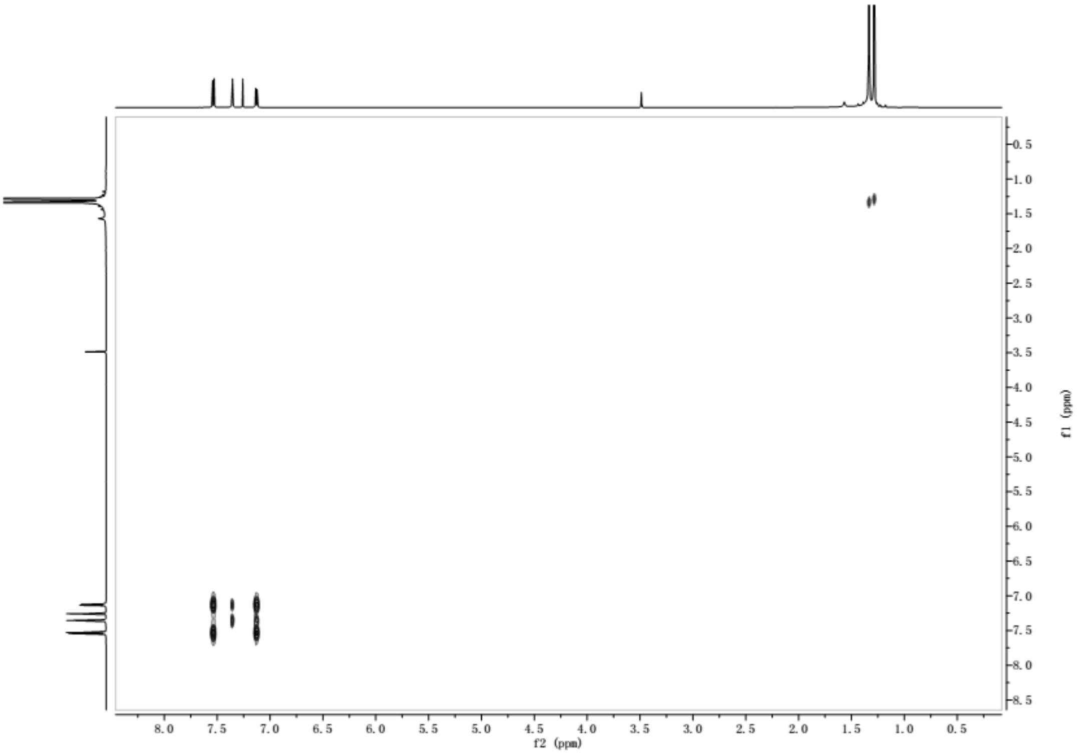

Figure S12. ^1H-^1H COSY spectrum of compound **2**.

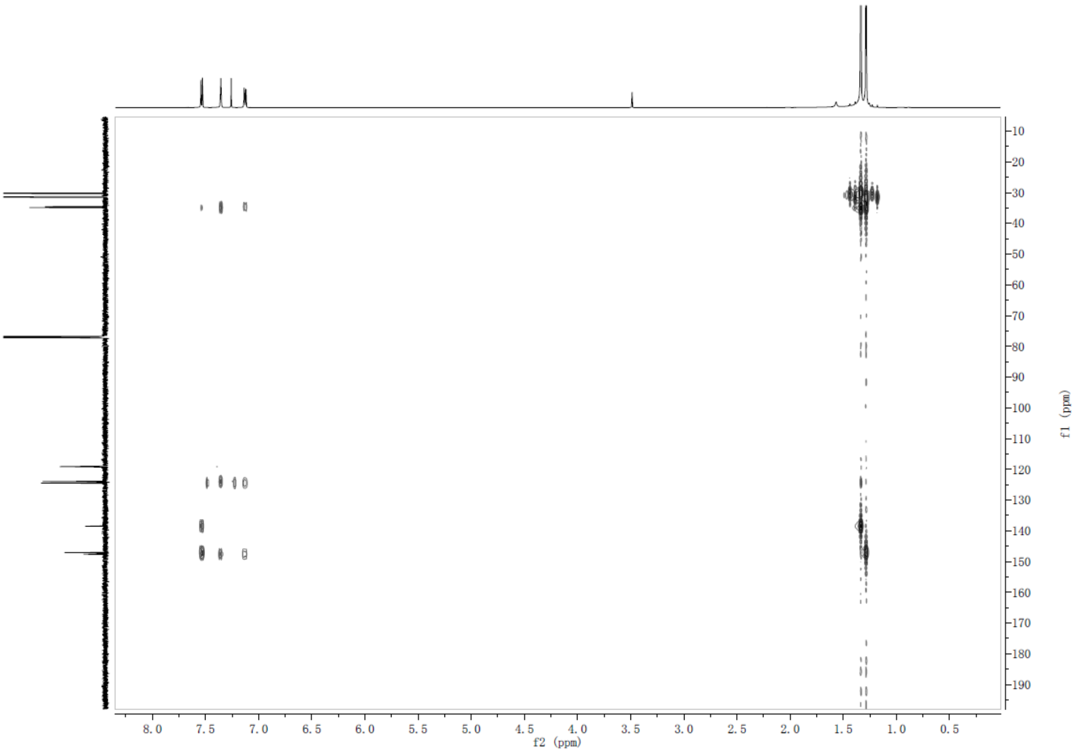

Figure S13. HMBC spectrum of compound **2**.

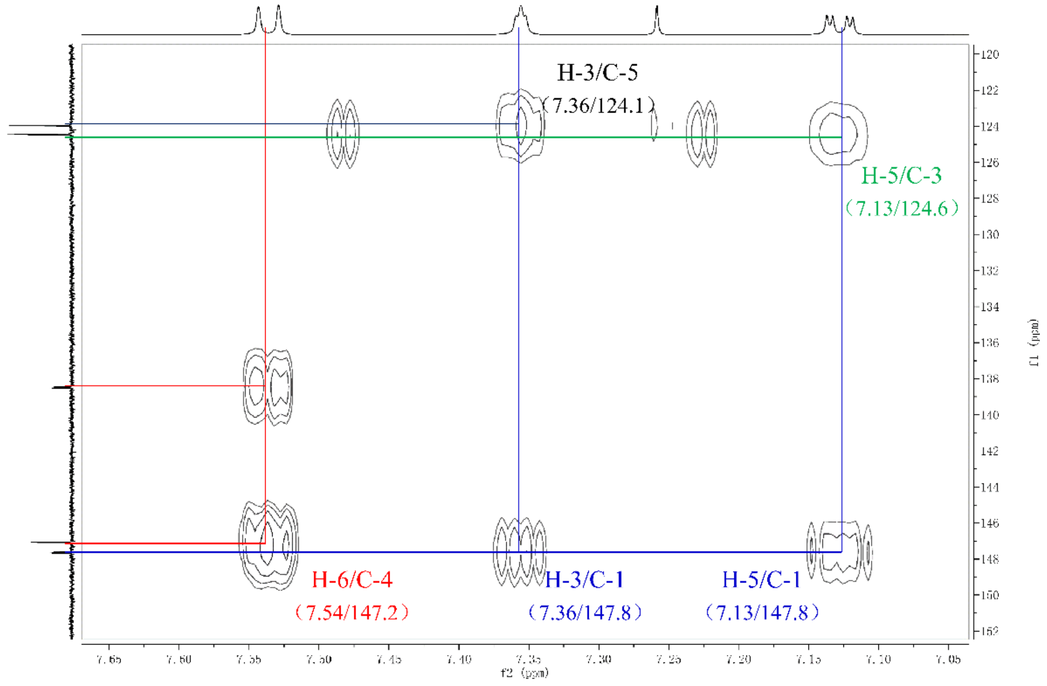

Figure S14 (I). The enlarged HMBC spectrum of compound **2** (I)

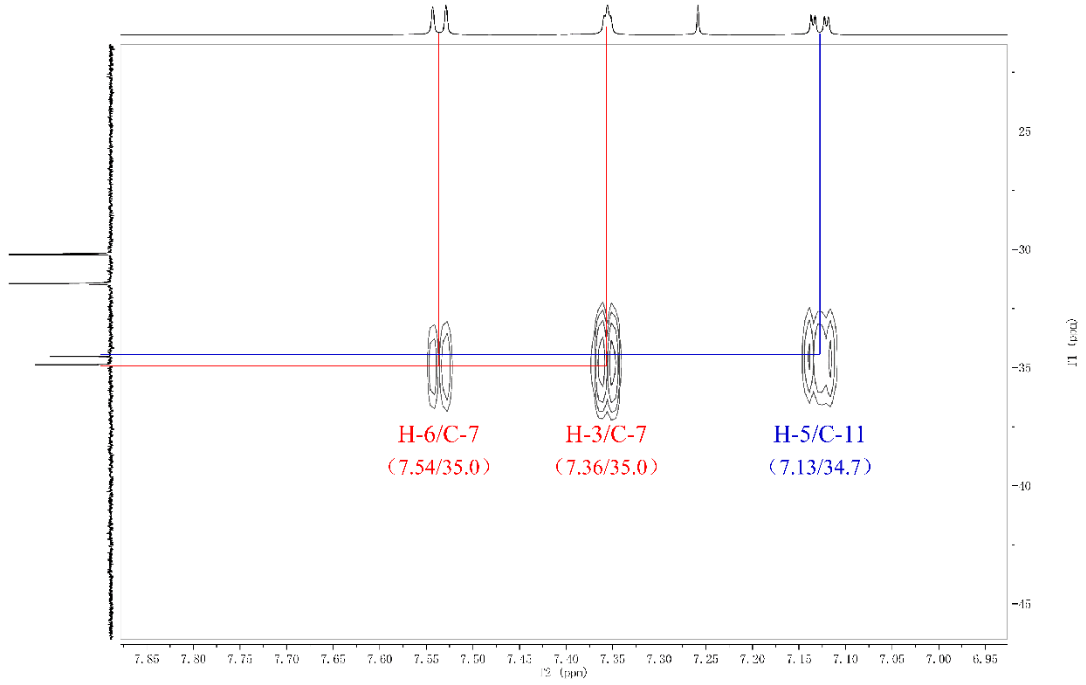

Figure S14 (II). The enlarged HMBC spectrum of compound **2** (II)

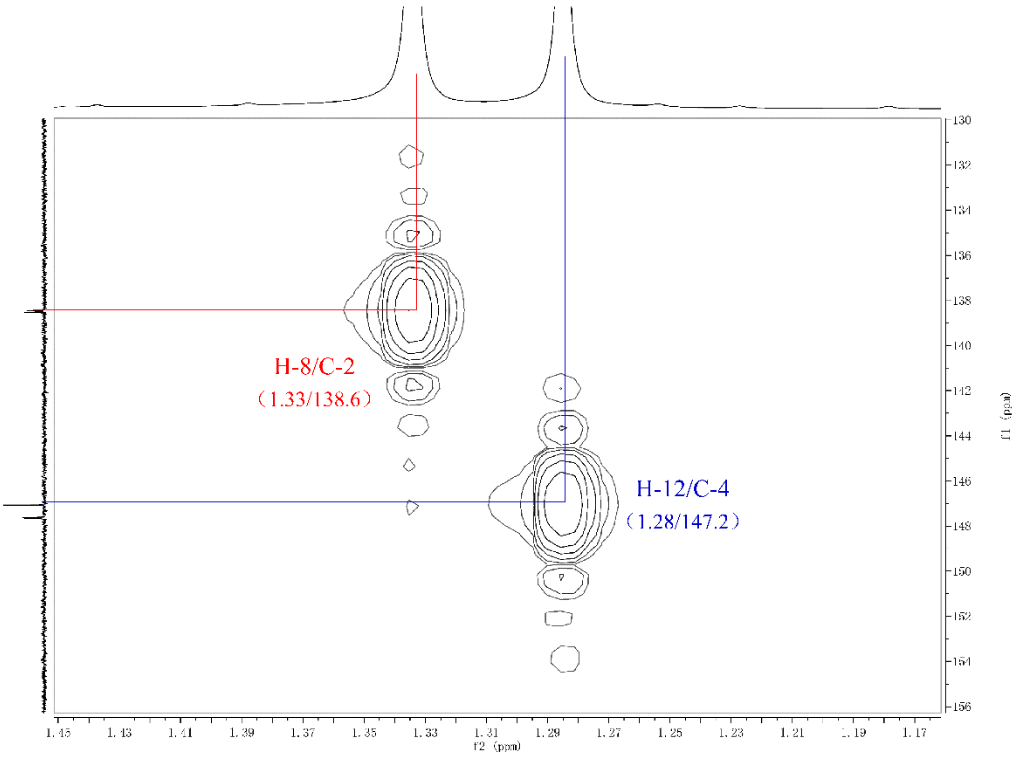

Figure S14 (III). The enlarged HMBC spectrum of compound **2** (III)

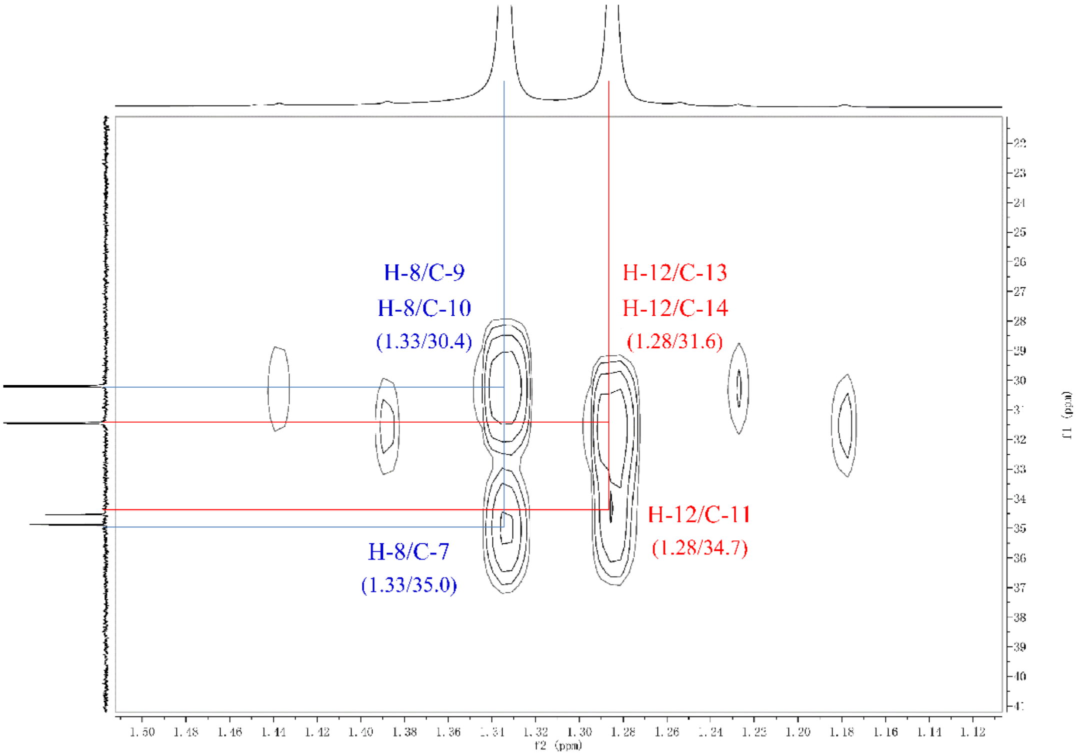

Figure S14 (IV). The enlarged HMBC spectrum of compound **2** (IV)

No.	**2** a)		2,4-DTBP b)		No.	**2'** c)	
	δ_H, mult (*J* in Hz)	δ_C	δ_H, mult (*J* in Hz)	δ_C		δ_H, mult (*J* in Hz)	δ_C
1 (1')		147.8 (147.8), C		152.0	2 (2')		147.6 (147.7)
2 (2')		138.6 (138.7), C		135.3	4 (4')		138.5 (138.5)
3 (3')	7.36, t (2.5)	124.6, CH	7.30, br s	124.2	3 (3')	7.36, d (2.5)	124.5
4 (4')		147.2, C		143.1	1 (1')		147.1
5 (5')	7.13, dd (8.6, 2.5)	124.1, CH	7.08, d (8.2)	123.7	5 (5')	7.13, dd (8.7, 2.5)	123.0
6 (6')	7.54, d (8.6)	119.3, CH	6.6, d (8.2)	116.1	6 (6')	7.54, d (8.7)	119.1
7 (7')		35.0, C		34.9	7 (7')		34.9
8/9/10 (8'/9'/10')	1.33, s	30.4, CH_3	1.42, s	29.8	12/13/14 (12'/13'/14')	1.29, s	30.2
11 (11')		34.7, C		34.4	11 (11')		34.5
12/13/14 (12'/13'/14')	1.28, s	31.6, CH_3	1.30, s	31.8	8/9/10 (8'/9'/10')	1.33, s	31.4

a) compounds **2, 2',** and **2,4-DTBP**, all measured in $CDCl_3$

b) **2,4-DTBP**, Journal of Chinese Medicinal Materials, **2019**, 07, 1541-1545; Journal of Ocean University of Qingdao, **1999**, 29(1): 53-56.

c) **2'**, Natural Product Research And Development, **2014**, 26(7): 987-989.

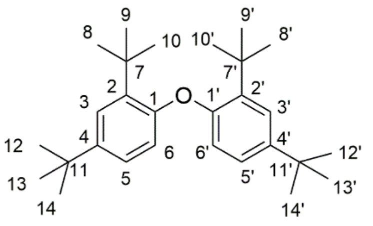

2 *revised*

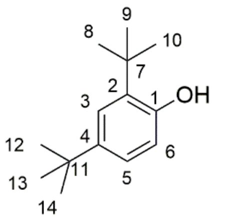

2,4-DTBP

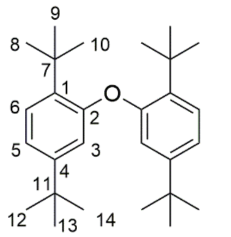

2' *originally proposed*

Figure S15. Structures and comparison of chemical shifts between compounds **2**, **2'**, and **2,4-DTBP** in $CDCl_3$

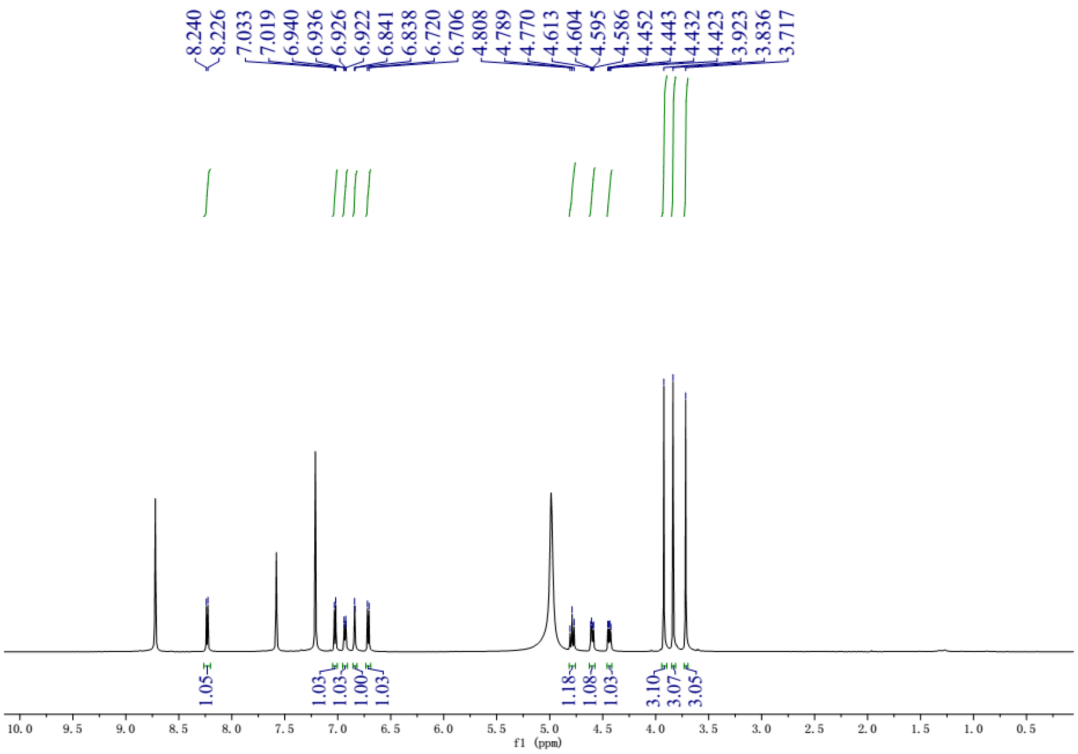

Figure S16. ^{1}H-NMR (600 MHz, C_5D_5N) spectrum of compound **3a**/**3b**.

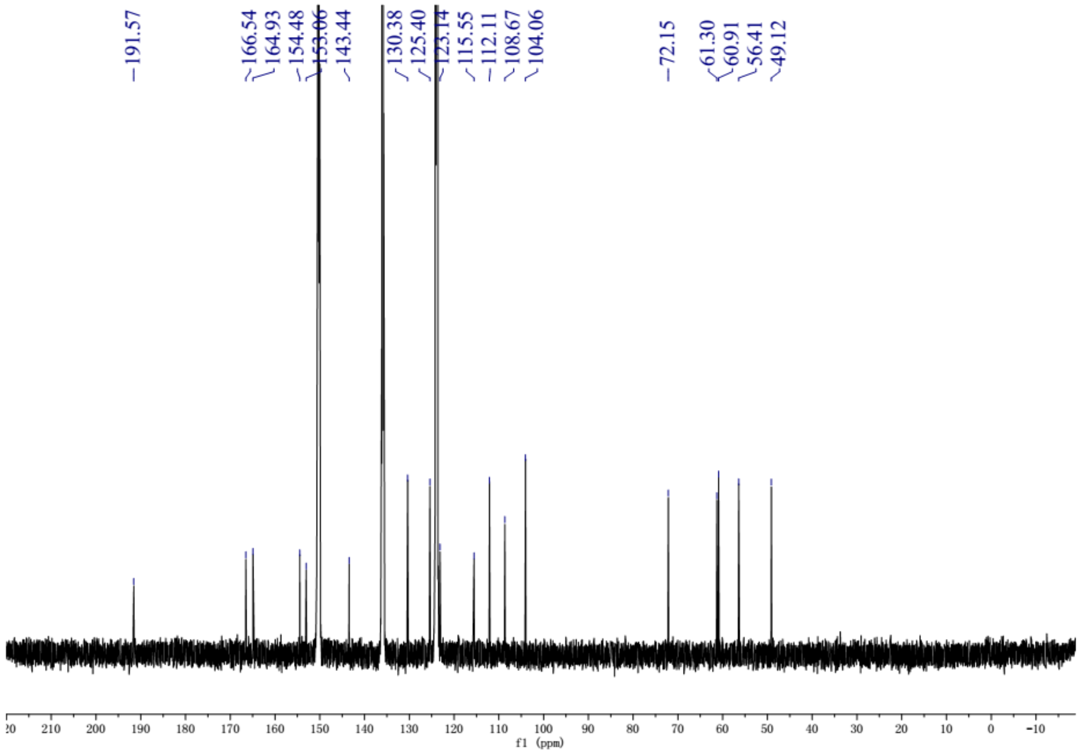

Figure S17. ^{13}C-NMR (150 MHz, C_5D_5N) spectrum of compound **3a**/**3b**.

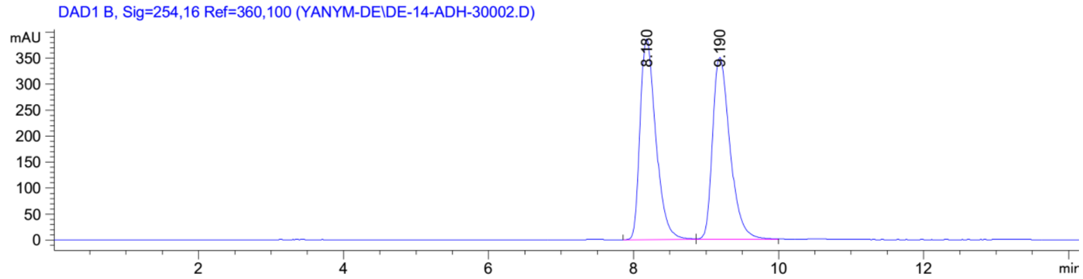

Figure S18. Chiral HPLC separation chromatogram of compound **3**

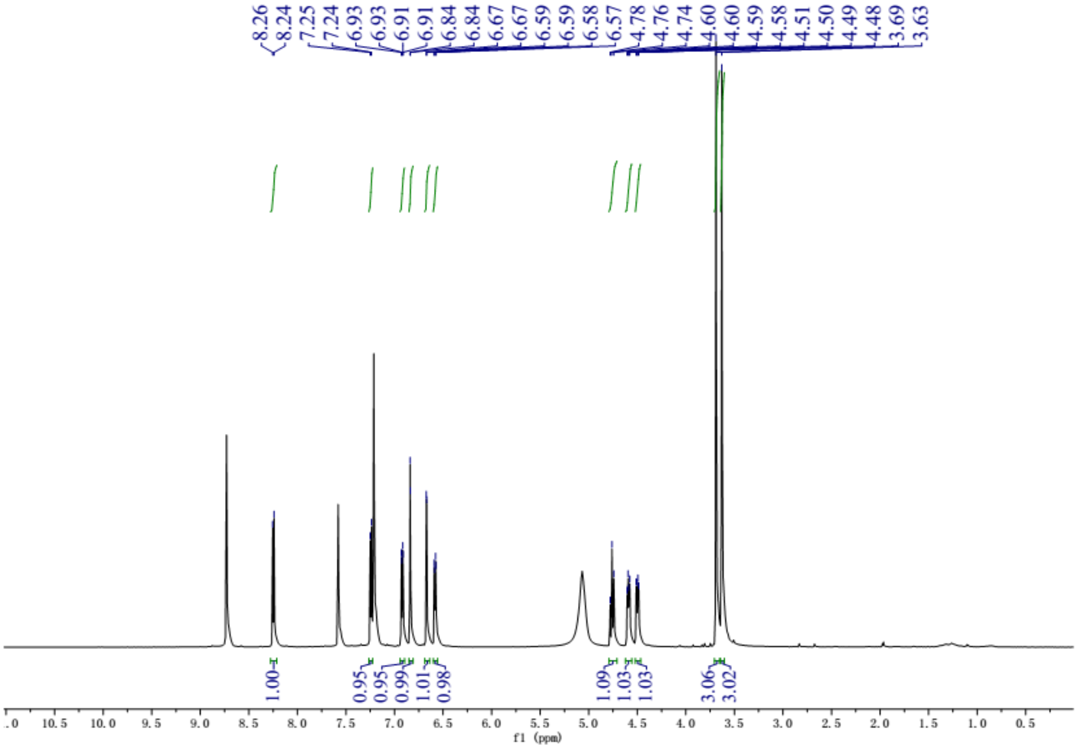

Figure S19. ^{1}H-NMR (600 MHz, C_5D_5N) spectrum of compound **4**.

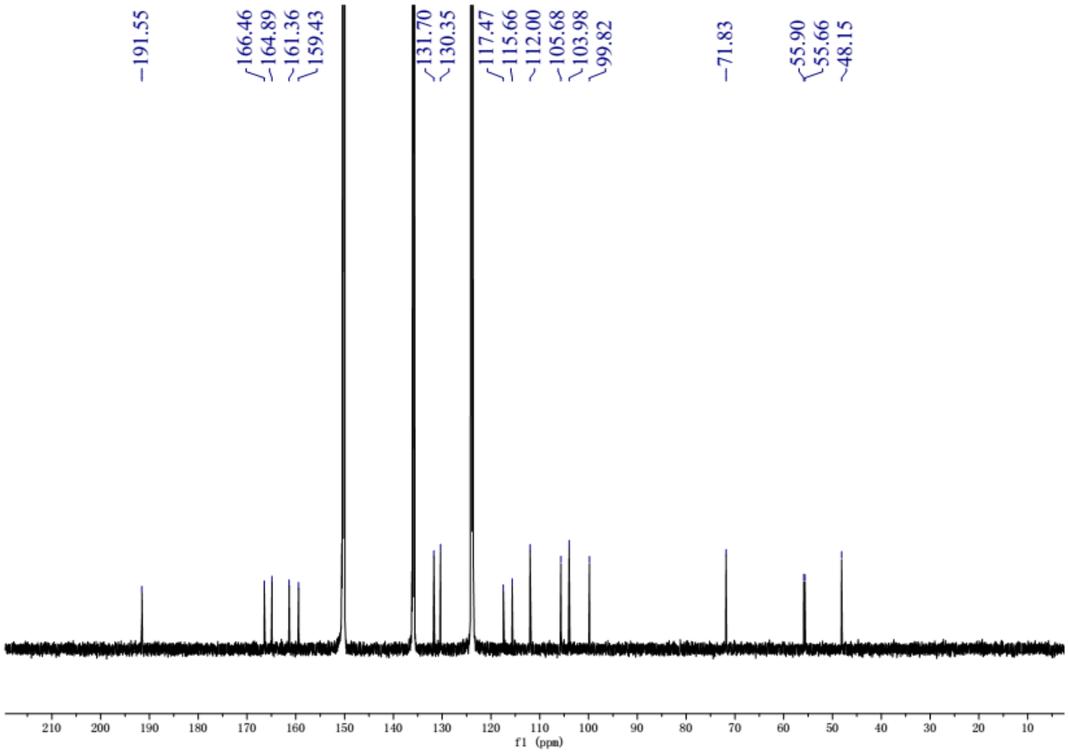

Figure S20. ^{13}C-NMR (150 MHz, C_5D_5N) spectrum of compound **4**.

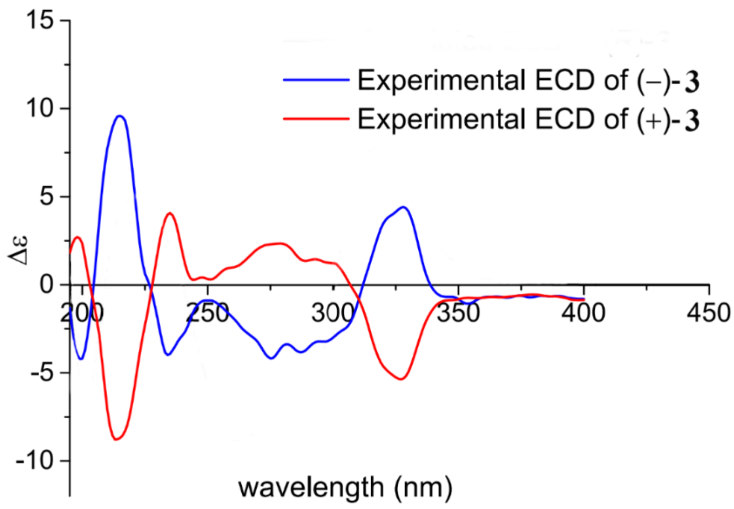

Figure S21. ECD spectrum of compound **3**

ECD calculation of compound 3

Computational methods

MMFF94 conformational search generated low-energy conformers within a 10 kcal/mol energy window were performed with CONFLEX.[1,2] Selected 2 most highest distribution conformers were further optimized by the density functional theory method at the B3LYP/6-31+g(d,p) level. The ECD calculations

was using TD-DFT-B3LYP/6-31+g(d,p) of theory on optimized geometries through the CPCM model (in MeOH). The calculated ECD curves were generated using SpecDis 1.61[3] with σ=0.30 ev, UV shift -10 nm. All the above calculations were carried out with the Gaussian 09 package of programs.[4]

References

(1) Goto, H.; Osawa, E.; J. Am. Chem. Soc. 1989, 111, 8950–8951.

(2) Goto, H.; Osawa, E.; J. Chem. Soc., Perkin Trans. 2, 1993, 187–198.

(3) Bruhn T.; Schaumlöffel A.; Hemberger Y.; Bringmann G.; *SpecDis*, version 1.61, University of Wuerzburg, Germany, **2013**.

(4) Frisch, M. J.; Trucks, G. W.; Schlegel, H. B.; Scuseria, G. E.; Robb, M. A.; Cheeseman, J. R.; Scalmani, G.; Barone, V.; Mennucci, B.; Petersson, G. A.; Nakatsuji, H.; Caricato, M.; Li, X.; Hratchian, H. P.; Izmaylov, A. F.; Bloino, J.; Zheng, G.; Sonnenberg, J. L.; Hada, M.; Ehara, M.; Toyota, K.; Fukuda, R.; Hasegawa, J.; Ishida, M.; Nakajima, T.; Honda, Y.; Kitao, O.; Nakai, H.; Vreven, T.; Montgomery, J. A.; Peralta, J. E.; Ogliaro, F.; Bearpark, M.; Heyd, J. J.; Brothers, E.; Kudin, K. N.; Staroverov, V. N.; Keith, T.; Kobayashi, R.; Normand, J.; Raghavachari, K.; Rendell, A.; Burant, J. C.; Iyengar, S. S.; Tomasi, J.; Cossi, M.; Rega, N.; Millam, J. M.; Klene, M.; Knox, J. E.; Cross, J. B.; Bakken, V.; Adamo, C.; Jaramillo, J.; Gomperts, R.; Stratmann, R. E.; Yazyev, O.; Austin, A. J.; Cammi, R.; Pomelli, C.; Ochterski, J. W.; Martin, R. L.; Morokuma, K.; Zakrzewski, V. G.; Voth, G. A.; Salvador, P.; Dannenberg, J. J.; Dapprich, S.; Daniels, A. D.; Farkas, O.; Foresman, J. B.; Ortiz, J. V.; Cioslowski, J.; Fox, D. J.

Gaussian 09, revision C.01. Gaussian, Inc.: Wallingford CT, **2010**.

Coordinates of computations (*R*)-**3**

Conformer 1

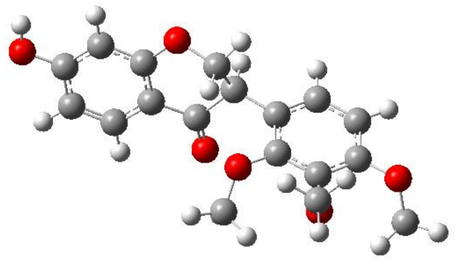

Standard orientation:

Center Number	Atomic Number	Atomic Type	Coordinates (Angstroms) X	Y	Z
1	6	0	5.366836	0.299100	0.070293
2	6	0	4.855214	0.876661	-1.109917
3	6	0	3.520746	0.695089	-1.420807
4	6	0	2.657795	-0.045064	-0.584106
5	6	0	3.193468	-0.597480	0.601542
6	6	0	4.544021	-0.434792	0.925734
7	6	0	1.268833	-0.317238	-0.981097
8	6	0	0.480166	-1.228821	-0.032839
9	6	0	1.017340	-1.100706	1.399586
10	8	0	2.441024	-1.330678	1.463931
11	8	0	0.790300	0.080473	-2.043673
12	8	0	6.686479	0.503823	0.330057
13	6	0	−1.023005	−1.047529	−0.085305
14	6	0	−1.857639	−2.138776	−0.353070
15	6	0	−3.246449	−2.006028	−0.378855
16	6	0	−3.831625	−0.770028	−0.102047
17	6	0	−3.018398	0.338560	0.199558
18	6	0	−1.616816	0.203446	0.173190
19	8	0	−0.797116	1.265467	0.482051
20	6	0	−0.862229	2.416606	−0.389261
21	8	0	−3.607018	1.547371	0.497907

22	6	0	−3.662957	1.852424	1.906003
23	8	0	−5.204015	−0.673971	−0.046807
24	6	0	−5.815548	0.090236	−1.103957
25	1	0	5.516985	1.444925	−1.754197
26	1	0	3.109806	1.114015	−2.333391
27	1	0	4.926502	−0.870228	1.843716
28	1	0	0.707141	−2.249249	-0.375750
29	1	0	0.809776	−0.103628	1.801238
30	1	0	0.574673	−1.854501	2.051172
31	1	0	6.946563	0.070940	1.156668
32	1	0	−1.412285	−3.108054	−0.558006
33	1	0	−3.886840	−2.853761	−0.599551
34	1	0	−0.626820	2.117831	−1.414782
35	1	0	−1.846557	2.886090	−0.343863
36	1	0	−0.100175	3.105234	−0.021757
37	1	0	−4.244432	1.092219	2.438427
38	1	0	−2.655719	1.916338	2.329548
39	1	0	−4.160019	2.820126	1.987324
40	1	0	−6.889535	0.063532	−0.913724
41	1	0	−5.461776	1.124504	−1.092999
42	1	0	−5.603039	−0.370181	−2.075666

Conformer 2

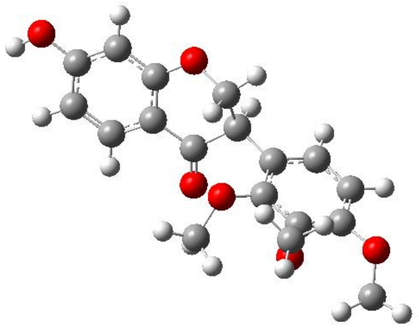

Standard orientation:

Center Number	Atomic Number	Atomic Type	Coordinates (Angstroms) X	Y	Z
1	6	0	5.368445	0.285590	0.087946
2	6	0	4.861560	0.871867	−1.090176
3	6	0	3.525460	0.695417	-1.405319
4	6	0	2.660476	−0.046437	−0.575443
5	6	0	3.193020	−0.606540	0.610156
6	6	0	4.541067	-0.449426	0.937435
7	6	0	1.271253	−0.312654	−0.977599
8	6	0	0.478636	−1.227213	−0.035826
9	6	0	1.012389	−1.106152	1.398638
10	8	0	2.434461	−1.342220	1.465769
11	8	0	0.796446	0.092229	−2.038999
12	8	0	6.672480	0.414409	0.455013
13	6	0	−1.024277	−1.045148	−0.091059
14	6	0	−1.858513	−2.135753	−0.362740
15	6	0	−3.247335	−2.003352	−0.388516
16	6	0	−3.832925	−0.768417	-0.107863
17	6	0	−3.020135	0.339544	0.197127
18	6	0	−1.618486	0.204845	0.171074
19	8	0	−0.799256	1.266069	0.484068
20	6	0	−0.863417	2.419366	−0.384323
21	8	0	−3.609264	1.547262	0.498850
22	6	0	−3.665174	1.848522	1.907756
23	8	0	−5.205353	−0.673097	−0.051952
24	6	0	−5.817691	0.092829	−1.107293
25	1	0	5.516177	1.445864	−1.739437
26	1	0	3.118715	1.120598	−2.316905
27	1	0	4.934463	−0.884195	1.849376
28	1	0	0.705995	−2.246366	−0.382334
29	1	0	0.807037	−0.109768	1.803316
30	1	0	0.564630	−1.860532	2.046069

31	1	0	7.155745	0.950617	−0.190675
32	1	0	−1.412777	−3.104320	−0.570202
33	1	0	−3.887419	−2.850640	−0.611785
34	1	0	−0.102994	3.107867	−0.013210
35	1	0	−0.625211	2.123528	−1.410069
36	1	0	−1.848337	2.887753	−0.340124
37	1	0	−4.246117	1.086544	2.438226
38	1	0	−2.657925	1.911903	2.331345
39	1	0	−4.162825	2.815695	1.991747
40	1	0	−6.891524	0.065833	−0.916249
41	1	0	−5.463862	1.127070	−1.094965
42	1	0	−5.605998	−0.365979	−2.079961
